# Depleting luminal cysteine with engineered bacteroides vulgatus alleviates experimental colitis by suppressing Th17 differentiation through an ATF6-dependent mechanism

**DOI:** 10.1007/s00011-026-02341-3

**Published:** 2026-09-05

**Authors:** Jianghao Wang, Zhe-Xian Tian, Shanwen Chen, Grace Y. Chen, Yi-Ping Wang, Pengyuan Wang

**Affiliations:** 1 Department of Gastrointestinal Surgery, Peking University First Hospital, Beijing, 100034 China; 2 The State Key Laboratory of Gene Function and Modulation Research, School of Life Sciences, Peking University, Beijing, 100871 China; 3 Department of Internal Medicine, Division of Gastroenterology, University of Michigan, Ann Arbor, USA; 4 Yazhouwan National Laboratory, Sanya, Hainan 572024 China; 5 Department of Emergency Surgery, Peking University People’s Hospital, Beijing, 100044 China

**Keywords:** Ulcerative colitis, Gut microbiota, Th17 differentiation

## Abstract

**Background:**

Ulcerative colitis (UC) is a chronic inflammatory bowel disease driven by dysregulated immune responses, particularly the aberrant activation of T helper 17 (Th17) cells. While microbiome-based therapies show promise, wild-type probiotics often lack specific mechanisms to target the metabolic and immunological drivers of inflammation.

**Methods:**

In this study, we engineered a cysteine-auxotrophic strain of *Bacteroides vulgatus* (BV1608) by chromosomally integrating the *E. coli cyuP* gene to enhance cysteine uptake. We evaluated its colonization capability, safety, and therapeutic efficacy in dextran sulfate sodium (DSS)-induced acute and chronic colitis murine models.

**Results:**

BV1608 exhibited superior colonization and cysteine assimilation compared to the wild-type strain. Oral administration of BV1608 significantly alleviated colitis symptoms, reduced pro-inflammatory cytokines, and restored intestinal barrier integrity. Mechanistically, BV1608 created a localized cysteine-restricted microenvironment in the gut and suppressed pathogenic Th17 differentiation. Under cystine-restricted conditions, ATF6 was activated in CD4⁺ T cells, and its inhibition partially restored IL-17A⁺ CD4⁺ T cell differentiation, indicating a functional role for ATF6. Meanwhile, cystine restriction was associated with increased BATF2 expression and enhanced ATF6 binding at the BATF2 promoter, suggesting BATF2 as a potential downstream node.

**Conclusion:**

Our findings demonstrate that metabolically engineered *B. vulgatus* BV1608 ameliorates colitis by coupling microbial cysteine sequestration with host immune modulation via the ATF6-dependent suppression of Th17 differentiation, while implicating BATF2-associated transcriptional regulation as a potential downstream mechanism. This study provides a novel synbiotic strategy for treating UC by targeting the immunometabolic interface.

**Supplementary information:**

The online version contains supplementary material available at https://doi.org/10.1007/s00011-026-02341-3.

## Introduction

Ulcerative colitis (UC), a major subtype of inflammatory bowel disease (IBD), is a chronic and relapsing inflammatory disorder of the colonic mucosa, posing a significant burden on patients’ quality of life globally [1]. The pathogenesis of UC is multifactorial, involving genetic predisposition, environmental triggers, dysregulated immune responses, and a disrupted gut microbiome [2–3]. Central to the disease pathology is the breakdown of intestinal immune homeostasis, characterized by the aberrant activation and differentiation of pro-inflammatory T helper 17 (Th17) cells, which drive excessive inflammation and tissue damage through the production of cytokines like IL-17 A [4]. Current therapies, including aminosalicylates, corticosteroids, and biological agents targeting TNF-α, primarily focus on suppressing the overarching immune response [5]. However, these strategies often yield inconsistent long-term efficacy and are associated with substantial side effects, highlighting the pressing need for novel therapeutic approaches that can precisely target the upstream drivers of intestinal inflammation.

Therapies aimed at modulating the gut microbiome, particularly using probiotics, have emerged as promising alternatives for managing UC by restoring microbial balance and reinforcing the intestinal barrier [6–8]. Among various commensals, *Bacteroides vulgatus*, a prevalent and abundant species in the human gut, has been reported to possess potential anti-inflammatory properties [9–10]. Nevertheless, the native and often inconsistent functionality of wild-type probiotics, coupled with their limited ability to withstand the inflammatory microenvironment and their lack of precise, mechanism-driven immunomodulatory actions, significantly restricts their therapeutic potency and clinical applicability [11]. A key challenge, therefore, lies in engineering probiotics to endow them with enhanced survival and targeted, therapeutic capabilities against specific pathogenic mechanisms, such as Th17-driven inflammation.

Recent advances in synthetic biology and metabolic engineering have enabled the rational design of probiotics with enhanced functionality and disease-specific targeting [12–14]. By introducing auxotrophies, engineered bacteria can be programmed to selectively colonize and function in inflamed niches, thereby achieving localized therapeutic effects with minimal off-target impact. Moreover, growing evidence highlights specific host signaling pathways that can be modulated by microbial metabolites [15].

In this study, we engineered a cysteine auxotrophic strain of *Bacteroides vulgatus* BV1608 to enhance its colonization and function in the inflamed colon—a microenvironment characterized by metabolic alterations. We hypothesized that this engineered strain would ameliorate colitis by restricting local cysteine availability and thereby modulating pathogenic Th17 differentiation. Our results demonstrate that metabolic reprogramming of *B. vulgatus* not only confers a colonization advantage but also suppresses Th17 differentiation in an ATF6-dependent manner. In addition, cystine restriction was associated with BATF2 upregulation and increased ATF6 binding at the BATF2 promoter, suggesting a potential ATF6–BATF2-associated transcriptional program. This work supports a synbiotic strategy that combines microbial metabolic engineering with host immune modulation, offering a promising and mechanistically informed approach for the treatment of UC.

## Materials and methods

### The construction of recombinant B. vulgatus BV1608

The recombinant *Bacteroides vulgatus* strain BV1608 was constructed to express the *E. coli cyuP* gene, which encodes a specific cysteine uptake enzyme. The construction involved a multi-step process to integrate the gene into the bacterial chromosome. An *IScepAp-cyuP* expression cassette was created by fusing the *Bacteroides IScepA* promoter to the *E. coli cyuP* coding sequence via nested PCR. This cassette was cloned into a transient vector for sequencing, with unique XbaI and KpnI restriction sites incorporated at its flanks. For chromosomal integration, the intergenic region between the *BVU_2794* and *BVU_2793* genes in *B. vulgatus* ATCC 8482 was selected as the target site. DNA fragments flanking this locus were spliced by nested PCR and cloned into the pLGB30 vector using Gibson Assembly, generating the intermediate plasmid pKUT1606 and introducing XbaI and KpnI sites into the chromosomal target. The final integration plasmid, pKUT1608, was generated by inserting the *IScepAp-cyuP* expression cassette into the XbaI-KpnI sites of pKUT1606. Plasmid pKUT1608 was transferred from an *E. coli* donor into *B. vulgatus* via biparental conjugation, enabling chromosomal integration of the expression cassette through double-crossover recombination.

### Culturing of bacteria

*Bacteroides vulgatus* was cultured on brain heart infusion (BHI) supplemented with hemin (5 mg/L) and vitamin K1 (2.5 mg/L) at 37 °C under anaerobic conditions. Fecal samples from healthy individuals and UC patients were thawed, vortexed, and cultured in BHIS rich medium anaerobically at 37 °C to an OD_600_ ≥ 1.0. To assess cysteine uptake, 100 µL of each culture was then transferred to 20 mL of M9S-cys limiting medium and incubated anaerobically at 37 °C to saturation [16]. Subsequently, the cysteine concentration in the supernatant was determined.

### Detection of cysteine levels

The cysteine concentration was determined using a commercial Cysteine Assay Kit (Solarbio, BC0180) according to the manufacturer’s instructions. Briefly, a standard curve was constructed using cysteine standards. Tissue samples or fecal samples were homogenized in the provided extraction buffer and centrifuged to obtain clear supernatants. Following the manufacturer’s protocol, the respective reagents were sequentially added to the samples. The mixture was incubated at room temperature for 15 min, and the absorbance was then measured at a wavelength of 600 nm.

### Cell culture

To prepare cystine-restricted medium, the modified RPMI 1640 medium (no glutamine, no methionine, no cystine) (Sigma-Aldrich) were supplemented with 0.1 mM methionine and 2 mM glutamine. Cells were cultured in medium with varying degrees of cystine restriction, supplemented with cystine at concentrations of 5, 50, and 200 µM, respectively.

### Animals and study design

The male C57BL/6J mice at the age of 8 weeks were bred inside the Animal Center of Peking University First Hospital. All mice experiments were approved by the Animal Ethics Committee of Peking University First Hospital. For acute colitis model, the mice were randomly assigned to four experimental groups: (1) Control: regular drinking water; (2) DSS: 3% (w/v) DSS solution in drinking water; (3) DSS + WT: 3% (w/v) DSS solution with oral gavage of BV-WT; (4) DSS + 1608: 3% (w/v) DSS solution with oral gavage of the BV1608. To induce acute colitis, mice were administered 3% (w/v) DSS solution (MP Biomedicals) in their drinking water for 7 days, during which body weight was monitored daily. At the same time, mice were daily administered sterile water, BV-WT or BV1608 (at a bacterial dose of 1 × 10^10^ CFU) via oral gavage. This was followed by a 3-day recovery period with normal water until day 10. On day 10, all mice were euthanized, their colons were measured for length, and colon tissue samples were collected for subsequent analysis. For chronic colitis model, the mice also were randomly assigned to four experimental groups: (1) Control: regular drinking water; (2) DSS: 1.5% (w/v) DSS solution in drinking water; (3) DSS + WT: 1.5% (w/v) DSS solution with oral gavage of BV-WT; (4) DSS + 1608: 1.5% (w/v) DSS solution with oral gavage of the BV1608. The mice were administered 1.5% (w/v) DSS solution for 7 consecutive days following which they were allowed to freely drink water for a further 14 consecutive days, this cycle was repeated three times. According to the group scheme, mice were administered the respective treatments by oral gavage every other day, and body weight was monitored in parallel. Then the mice were euthanized, the length of colon was measured and the colon tissues were collected for subsequent analysis.

### Disease activity index score

Disease activity in the DSS-induced colitis model was quantified using an established Disease Activity Index (DAI) [17]. The DAI score (0–4) was derived from the average of three parameters: body weight loss percentage, stool consistency, and fecal bleeding.

### H&E staining and analysis

Tissue sections were deparaffinized, rehydrated, and stained with Harris hematoxylin. After differentiation and bluing, eosin counterstaining was performed. The sections were then dehydrated, cleared in xylene, and mounted with neutral balsam for microscopic observation. Three representative fields of view per sample were selected for imaging, followed by quantitative assessment and statistical analysis. The H&E staining was scored according to a previously established scoring system [18].

### Immunohistochemistry

Following deparaffinization in xylene and graded ethanol rehydration, antigen retrieval was performed by microwaving in EDTA buffer. Endogenous peroxidase activity was blocked, and sections were incubated with goat serum before overnight treatment with anti-MPO antibody (Proteintech) at 4 °C. After PBS washes, sections were incubated with HRP-conjugated secondary antibody, developed with DAB, and counterstained with hematoxylin.

### Immunofluorescence (IF) staining

Paraffin-embedded tissue sections were deparaffinized, rehydrated, and subjected to antigen retrieval in EDTA buffer (pH 8.0) by microwave heating. After cooling and PBS washing, sections were blocked with goat serum and incubated overnight at 4 °C with primary antibody. Following PBS washes, sections were incubated with a species-matched fluorescent secondary antibody for 1 h at room temperature in the dark, then counterstained with DAPI. After final washes, sections were mounted with anti-fade medium and imaged by fluorescence microscopy.

### Enzyme-linked immunosorbent assay (ELISA)

The levels of TNF-α, IL-6, and IL-1β were measured using commercial ELISA kits (Elabscience) according to the manufacturer’s instructions with slight modifications. Briefly, standards, blanks, and samples were incubated for 90 min at 37 °C, followed by sequential addition of a biotinylated detection antibody (1 h, 37 °C), HRP conjugate (30 min, 37 °C), and substrate solution (15 min). After stopping the reaction, the absorbance was read at 450 nm.

### Assessment of intestinal permeability

We evaluated intestinal permeability using fluorescein isothiocyanate-dextran (FITC-dextran, Sigma-Aldrich). Mice were fasted for 12 h and then gavaged with FD4 (500 mg/kg). After 4 h, serum was obtained from blood samples. The fluorescence intensity of FD4 in the serum was measured at 492/520 nm, and concentrations were calculated using a standard curve. Furthermore, serum LPS levels were determined with a mouse-specific ELISA kit to assess endotoxemia.

### Serum analysis

Serum was separated from blood samples by clotting at room temperature for 1 h followed by centrifugation at 3000 rpm for 15 min. The levels of renal function indices (creatinine, CREA, and urea, UREA) and liver function parameters (alanine aminotransferase, ALT, and aspartate aminotransferase, AST) were assessed using commercial assay kits (Beijing Beijian Xinchuangyuan Biological Technology, China) in accordance with the manufacturer’s protocols to evaluate the potential toxicity of engineered Bacteroides strain 1608 on hepatic and renal functions.

### Western blotting

Proteins isolated from proximal colon mucosa using RIPA buffer (Beyotime) were quantified by BCA assay. Subsequent to SDS-PAGE and transfer to PVDF membranes, blocking was carried out with 5% non-fat milk in TBST. Membranes were then probed with primary antibodies overnight at 4 °C and HRP-conjugated secondaries for 1 h at room temperature. Protein bands were visualized via ECL. Primary antibodies were included that mouse anti-Occludin antibody (Invitrogen; 33-1500), rabbit anti-E-cadherin antibody (abcam, ab231303), rabbit anti-BATF2 antibody (Invitrogen; PA5-37138) and rabbit anti-ATF6 antibody (CST; 65880).

### Isolation of lamina propria mononuclear cells

Lamina propria Mononuclear Cells (LPMCs) were isolated from mouse colon using a combined mechanical and enzymatic dissociation protocol [19]. Briefly, the colon was dissected, opened longitudinally, and cut into 0.5 cm segments. Tissues were washed thoroughly in HBSS containing 2.5% FBS and antibiotics, followed by sequential incubation in 1 mM DTT and 1 mM EDTA solutions at 37 °C with continuous stirring. The remaining tissue was then digested in pre-warmed solution containing 400 U/mL collagenase type III and 10 µg/mL DNase for approximately 2 h at 37 °C until tissue fragments were fully dissolved. The resulting cell suspension was filtered through a 70 μm strainer. Lymphocytes were further purified by density gradient centrifugation using a 40%/75% Percoll system.

### Human peripheral blood CD4⁺ T cells isolation and polarization

Naïve CD4⁺ T cells were isolated from human Peripheral blood mononuclear cells (PBMCs) using Ficoll density gradient centrifugation, followed by purification with CD4 Nanobeads (BioLegend). The cells were cultured in complete RPMI-1640 medium (supplemented with 10% FBS and 1% Penicillin-Streptomycin) and stimulated with CD3/CD28 T Cell Activator (Stemcell). The cells were then induced to differentiate into Th17 cell subsets with a cytokine cocktail containing IL-1β (10ng/ml, BioLegend), IL-6 (50ng/ml, PeproTech^®^), IL-23 (10ng/ml, PeproTech^®^) anti-IL-4 antibody (10 µg/ml, BioLegend) and anti-IFN-γ antibody (10 µg/ml, BioLegend).

### Flow cytometry

Cultured Naïve CD4⁺ T cells or cells isolated from mouse spleen, colon LP or PBMCs were washed and resuspended in ice-cold flow cytometry staining buffer (FACS buffer; PBS with 1% FBS). For surface staining, the manufacturer’s recommended concentration of the appropriate antibodies was added to the cells and incubated in the dark at 4℃ for 40 min.; For intracellular staining, the cells were fixed and permeabilized sequentially in fixation/permeabilization buffer, then permeabilization buffer, the appropriate antibodies were added, and the cells were incubated at RT for 30 min. After removal of unbound antibodies by washing, cells were resuspended in 300 µl PBS and analyzed.

### Chromatin immunoprecipitation-qPCR (ChIP-qPCR)

The entire procedure was performed according to the instructions of the Pierce Magnetic ChIP Kit (Cat: #26157, Thermo Scientific). Briefly, cells were crosslinked with 1% formaldehyde, quenched with glycine, and lysed. Isolated nuclei were digested with MNase and sonicated to shear chromatin. The chromatin was immunoprecipitated using target-specific antibodies and Protein A/G magnetic beads. After washing, bound complexes were eluted and reverse crosslinked with Proteinase K. DNA was purified using the supplied clean-up columns and analyzed by qPCR. The primer sequences for the BATF2 promoter: Site1, 1 F: CACTAATCGATCATGGTGTGGC and 1R: TGATAGGTGATGGTGGCTGG; Site2, 2 F: TTGCCATTCTTGTGATACCACATC and 2R: AGTAATTCCTGTCCAAGAACAGC; Site3, 3 F: GGAAGGCCAGTTCATGTTAGG and 3R: CCTAAGGAAGTGGCAGCTC.

### Cytoplasmic/nuclear protein extraction

Nuclear and cytoplasmic proteins were isolated for further analysis using NE-PER Nuclear and Cytoplasmic Extraction Reagents (Cat: #78833, Thermo Scientific) according to the manufacturer’s instructions. Briefly, harvested cell pellets were thoroughly resuspended in ice-cold Cytoplasmic Extraction Reagent I (CER I) and incubated on ice, followed by the addition of CER II and brief vortexing. After centrifugation, the cytoplasmic extract was transferred to a pre-chilled tube. The insoluble pellet containing nuclei was then resuspended in Nuclear Extraction Reagent (NER) and vortexed periodically during a 40-minute incubation on ice. The nuclear extract was finally collected as the supernatant following a high-speed centrifugation step.

### RNA sequencing

Total RNA libraries derived from CD4⁺ T cells were sequenced on an Illumina platform (150 bp paired-end). Clean reads were mapped to the reference genome using HISAT2 (v2.0.5) and quantified as FPKM via featureCounts (v1.5.0-p3). Differentially expressed genes were identified using DESeq2 (v1.20.0) with an adjusted P-value < 0.05. Functional enrichment (GO, KEGG) and GSEA analyses were performed using clusterProfiler (v3.8.1).

### 16 S rRNA gene sequencing and analysis

Fresh fecal samples were collected in sterile microcentrifuge tubes, snap-frozen in liquid nitrogen, and stored at –80 °C. Microbial genomic DNA was extracted from fecal samples using the HiPure Stool DNA Kit (Magen, Guangzhou, China) following the manufacturer’s instructions. The V3–V4 hypervariable regions of the bacterial 16 S rRNA gene were amplified using primers 338 F (5’-ACTCCTACGGGAGGCAGCAG-3’) and 806R (5’-GGACTACHVGGGTWTCTAAT-3’). PCR amplicons were purified, quantified, and paired-end sequenced (PE250) on an Illumina platform. Raw reads were quality-filtered, merged, and clustered into Operational Taxonomic Units (OTUs) at 97% similarity using VSEARCH (v1.9.6). Taxonomic annotation was performed using the RDP classifier against the SILVA 138 database. Alpha diversity indices (Shannon, Chao1) were calculated to evaluate species richness and diversity. Beta diversity was visualized via Principal Coordinate Analysis (PCoA) based on Bray-Curtis or UniFrac distances. Differentially abundant taxa between groups were identified using Linear Discriminant Analysis Effect Size (LEfSE).

### Statistical analysis

Results are presented as mean ± standard error (S.E.M.). Comparisons between two groups were performed using unpaired two-sample t-tests. For multiple comparisons involving more than two groups, one-way ANOVA followed by Tukey’s multiple comparison tests was employed.* P* values < 0.05 were deemed statistically significant. All statistical analyses were conducted using GraphPad Prism 10.0.

## Results

### Targeted engineering of cysteine-auxotrophic *B. vulgatus* BV1608 for enhanced cysteine uptake

**Fig. 1:**
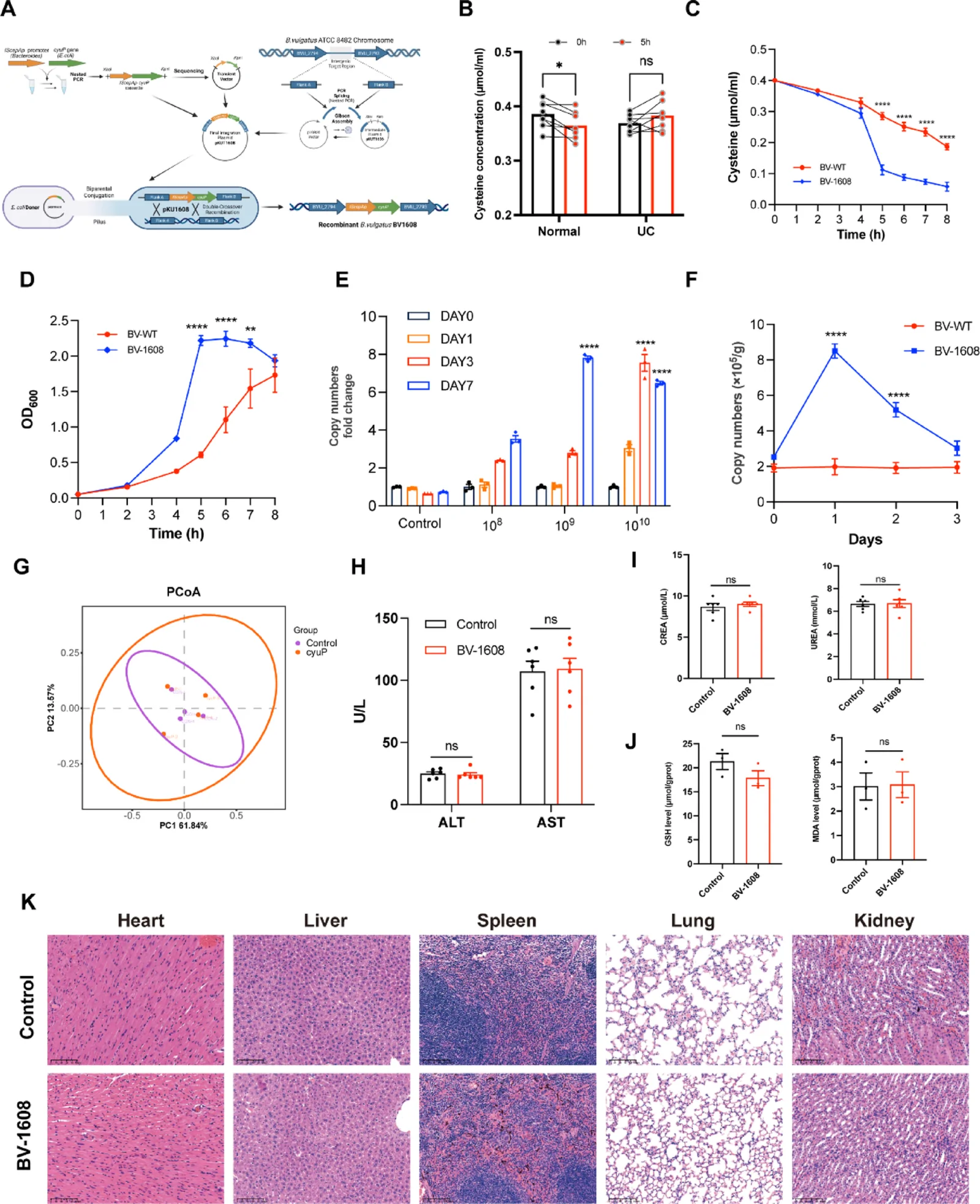
Construction, biological characterization, and safety assessment of the engineered *B. vulgatus* BV1608. **A** Schematic diagram of the engineered *B. vulgatus* BV1608 construction. **B** Cysteine levels in culture supernatants after 5-hour incubation of fecal bacteria from healthy donors and UC patients. **C–D** Cysteine utilization and growth curves of wild-type and BV1608 in a cysteine-limited medium (0.4 mM). **E** qPCR quantification of *B. vulgatus* BV1608 in fecal samples from mice after 7-day consecutive gavage with different dosages. (Control:0; 10^8^:10^8^ CFU/ml; 10^9^:10^9^ CFU/ml; 10^10^:10^10^ CFU/ml). **F** Colonization dynamics of engineered *Bacteroides* in the mouse gut following a single oral gavage (10^10^ CFU/ml). **G** PCoA plot of the gut microbiota based on the Bray–Curtis metric between control and BV1608. **H–I** Serological indices of liver and kidney function. (J) Quantitative analysis of GSH and MDA levels in colonic tissue homogenates. **K** Representative images of H&E-stained heart, liver, spleen, lung, kidney. Data are presented as mean values ± SEM. Statistical analysis was performed using unpaired Student t test in 2 groups. Statistical analysis was performed using one-way or two-way ANOVA in multiple groups. *P < 0.05, **P < 0.01 and ****P < 0.0001 (*n* = 3 biologically independent samples for** C–F** and J, *n* = 6 biologically independent samples for H and I, *n* = 8 biologically independent samples for B). UC ulcerative colitis, PCoA principal coordinates analysis, GSH glutathione, MDA malondialdehyde, H&E hematoxylin and eosin

We engineered the wild-type *Bacteroides vulgatus* strain (BV-WT) to allow for the exogenous expression of the *cyuP* gene, which encodes the CyuP protein—a functional cysteine uptake enzyme [20]. This modification was implemented with the aim of conferring an enhanced cysteine assimilation capability and the construction of *B. vulgatus* BV1608 was carried out as illustrated in the schematic diagram in Fig. 1A. Fecal samples were collected from patients with UC and healthy controls. Following a 5-hour incubation to reach bacterial saturation, we observed that the fecal microbiota of healthy controls exhibited a significantly higher capacity for cysteine utilization compared to that of UC patients (Fig. 1B). To compare the cysteine uptake activity between the BV1608 and BV-WT, both strains were cultured in a defined liquid medium. Bacterial growth kinetics and the residual cysteine concentration in the culture supernatant were monitored over an 8-hour period (Fig. 1C–D). The first four hours were characterized by a lag phase with similar, slow growth rates and comparable cysteine consumption profiles between the two strains. During the subsequent four-hour period, the growth profiles of the two strains diverged markedly. Specifically, at the 5-hour time point, the growth rate of the engineered strain accelerated significantly, with a sharp decrease in the cysteine concentration in the culture medium.

To investigate the in vivo colonization capacity of the engineered strain, C57BL/6 N mice were orally gavaged with varying concentrations (CFU/ml) of the bacterial suspension every other day for a total of three times. The engineered strain colonized most effectively at a concentration of 10^10^ CFU/mL (Fig. 1E). The metabolic timeline of the bacteria in the intestinal tract after a single oral dose was determined to be approximately three days for complete clearance (Fig. 1F). 16 S rRNA gene sequencing analysis showed that administration of the BV1608 did not significantly affect the diversity of the gut microbiota (Fig. 1G). Subsequently, the mice were subjected to 7 consecutive days of gavage to determine the systemic safety. Compared to the control group, the BV1608 gavage group showed no significant differences in liver and kidney function parameters (Fig. 1H-I). Furthermore, glutathione and malondialdehyde levels were quantified in murine colonic tissue homogenates to test the oxidative stress and epithelial redox homeostasis. We found that no statistically significant differences were observed between the control group and the BV-1608 group (Fig. 1J). H&E staining revealed no damage to the liver, kidney, spleen, heart, and lung, manifested by all examined sections displaying no abnormalities (Fig. 1K).

### Oral administration of *B. vulgatus* BV1608 alleviated DSS-induced acute colitis injury

**Fig. 2:**
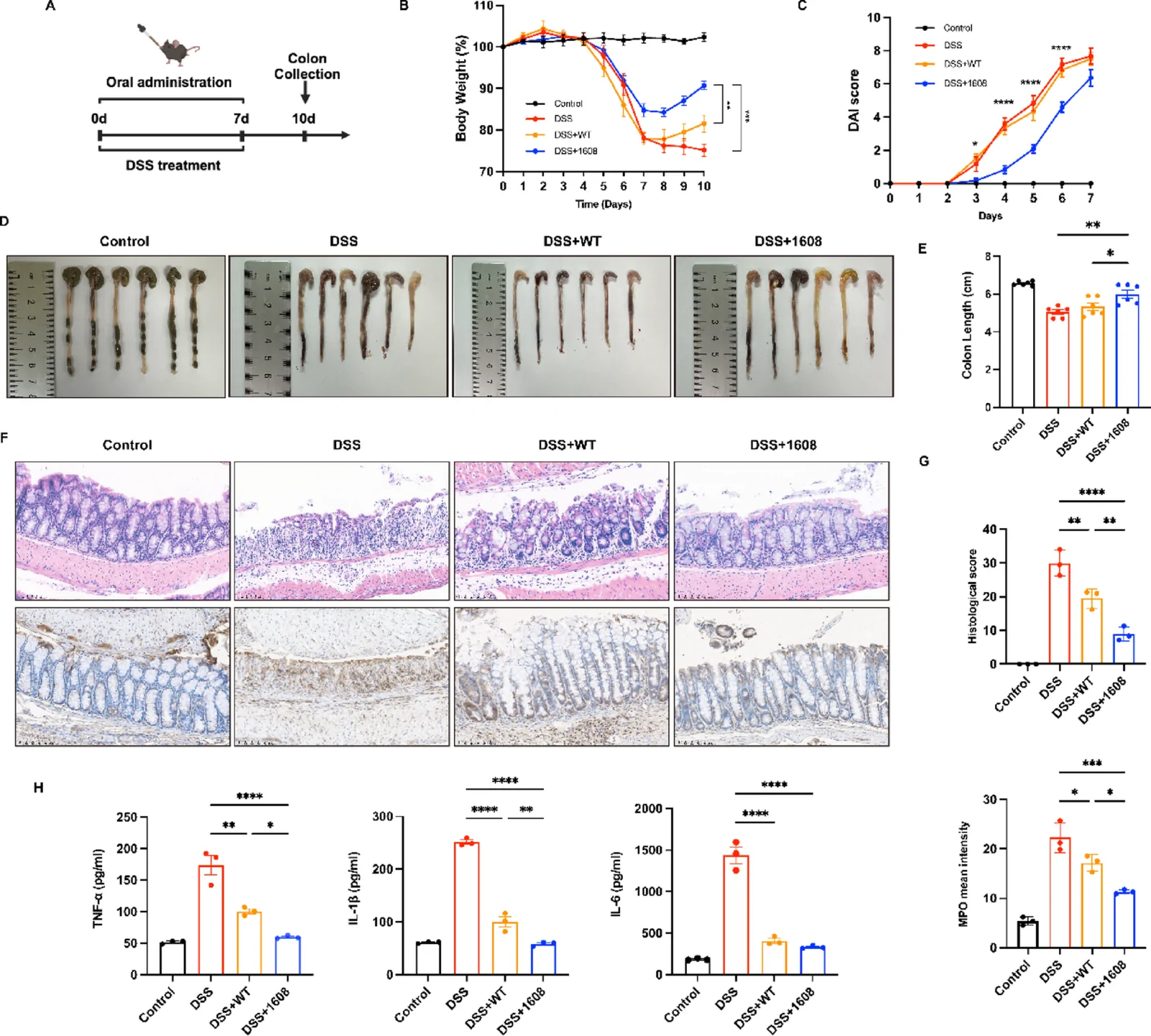
Oral administration of BV1608 alleviates symptoms and inflammation in DSS-induced acute colitis mice. **A** Experiment design of the therapeutic strategy using engineered bacteria to intervene in a mouse model of acute colitis. **B–C** Body weight change and DAI score curves. **D–E** Macroscopic colon images and quantitative statistical analysis of colon length. **F** Representative images of colon sections stained with H&E and for MPO by immunohistochemistry. (Scale bar: 100 μm). **G** Average macroscopic colon scores and MPO mean intensity were calculated for each group. **H** ELISA was used to measure the concentrations of TNF-α, IL-1β, and IL-6 in mouse ocular serum. Data are presented as mean values ± SEM. Statistical analysis was performed using one-way or two-way ANOVA. *P < 0.05, **P < 0.01, ***P < 0.001, ****P < 0.0001 (*n* = 6 biologically independent samples for B, C and E, *n* = 3 biologically independent samples for** G** and** H**). DAI disease activity index, H&E hematoxylin and eosin, MPO myeloperoxidase, ELISA Enzyme-Linked Immunosorbent Assay

To investigate the therapeutic effect of the BV1608 on IBD, an acute colitis model was established in mice using 3% DSS (Fig. 2A). Body weight changes were similar across groups in the first 7 days. Following the transition to normal water, the oral BV1608 group demonstrated the most marked recovery in body weight, with the BV-WT group showing an intermediate effect. Statistically significant differences were confirmed for both treated groups versus the DSS group (Fig. 2B). The BV1608-treated group demonstrated a significant reduction in the DAI score, indicating the most attenuated intestinal pathological changes (Fig. 2C). Measurement of colon length revealed that both the wild-type and BV1608 strains significantly alleviated DSS-induced colon shortening. Moreover, the protective effect of the BV1608 strain was markedly superior to that of the BV-WT (Fig. 2D–E). Consistent with previous findings, assessment of histopathological damage (H&E staining) and neutrophil infiltration (MPO activity) confirmed that the BV1608 strain was the most effective treatment in ameliorating colitis (Fig. 2F–G). Finally, analysis of key inflammatory cytokines (TNF-α, IL-1β, IL-6) in serum showed that BV1608 strain produced a broader and stronger suppression of the inflammatory response than the BV-WT strain, which only partially reduced the levels of TNF-α and IL-1β (Fig. 2H).

### Oral administration of *B. vulgatus* BV1608 alleviated DSS-induced chronic colitis injury

**Fig. 3:**
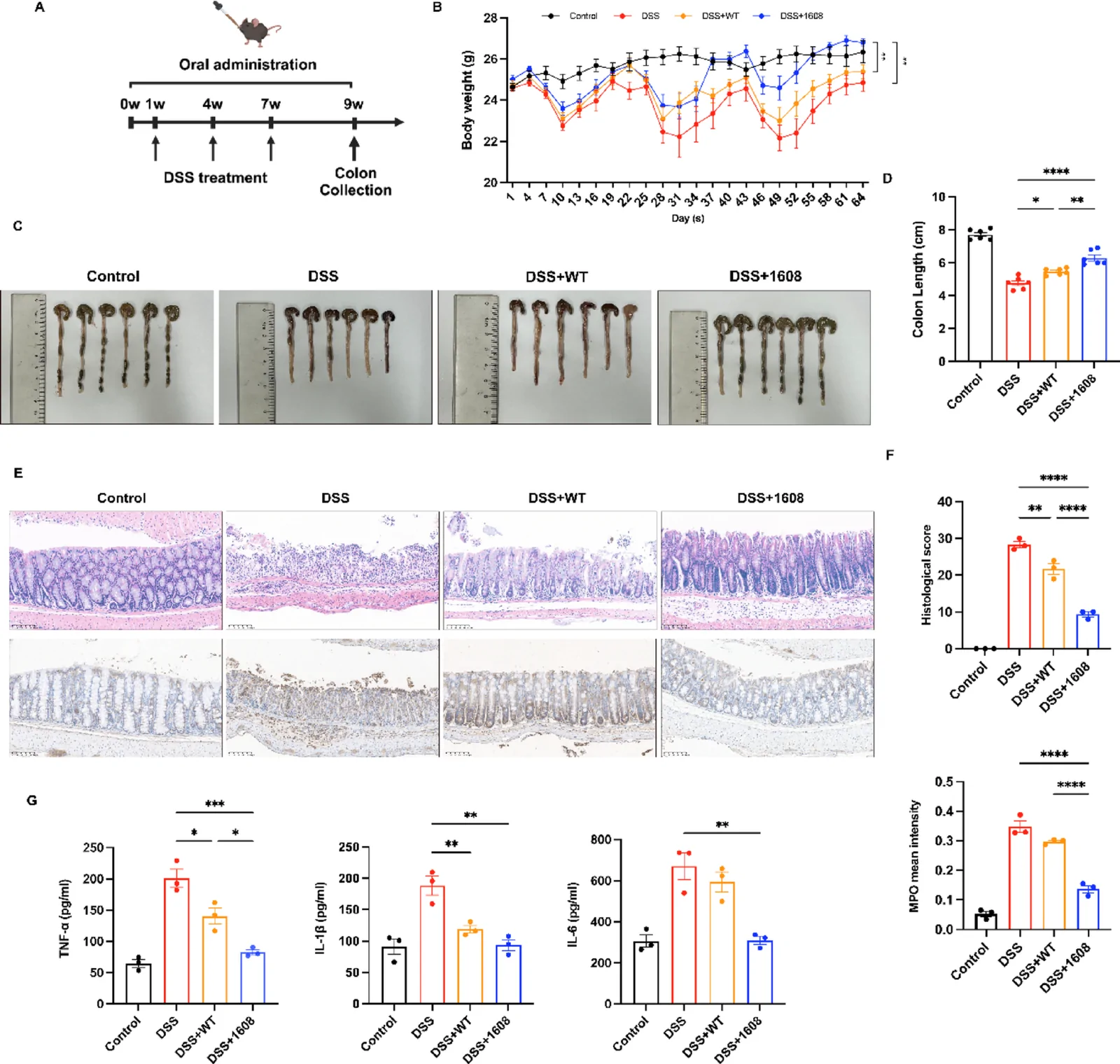
Therapeutic efficacy of BV1608 in a murine model of DSS-induced chronic colitis. **A** Experiment design of the therapeutic strategy using engineered bacteria to intervene in a mouse model of chronic colitis. **B** Body weight change curve. **C–D** Macroscopic colon images and quantitative statistical analysis of colon length. **E** Representative images of colon sections stained with H&E and for MPO by immunohistochemistry. (Scale bar: 100 μm). **F** Average macroscopic colon scores and MPO mean intensity were calculated for each group. **G** The levels of TNF-α, IL-1β and IL-6 in the mouse ocular blood serum measured via ELISA. Data are presented as mean values ± SEM. Statistical analysis was performed using one-way or two-way ANOVA. *P < 0.05, **P < 0.01, ***P < 0.001, ****P < 0.0001 (*n* = 6 biologically independent samples for B and D, *n* = 3 biologically independent samples for F and G). **H&E** hematoxylin and eosin, MPO myeloperoxidase, ELISA Enzyme-Linked Immunosorbent Assay

After observing that BV1608 can alleviate acute colitis injury, we further investigated the therapeutic efficacy on chronic colitis. Following the experimental timeline outlined in Fig. 3A, we established a mouse model of chronic colitis. Subsequent statistical analysis revealed a significant reduction in both body weight and colon length in the model group. Notably, these alterations were markedly ameliorated following oral administration of BV1608 (Fig. 3B–D). Furthermore, histopathological analysis including H&E staining and MPO immunohistochemical scoring demonstrated that BV1608 significantly attenuated chronic intestinal mucosal damage and neutrophil infiltration induced by DSS (Fig. 3E–F). Finally, regarding the inflammatory cytokines, the serum levels of pro-inflammatory factors, including TNF-α, IL-1β and IL-6 were significantly reduced following BV1608 treatment (Fig. 3G). This observation is consistent with the alleviation of chronic colitis injury, further supporting the therapeutic efficacy of oral BV1608 administration.

### Reduction of intestinal cysteine and restoration of barrier function by oral *B. vulgatus* BV1608 in colitis

**Fig. 4:**
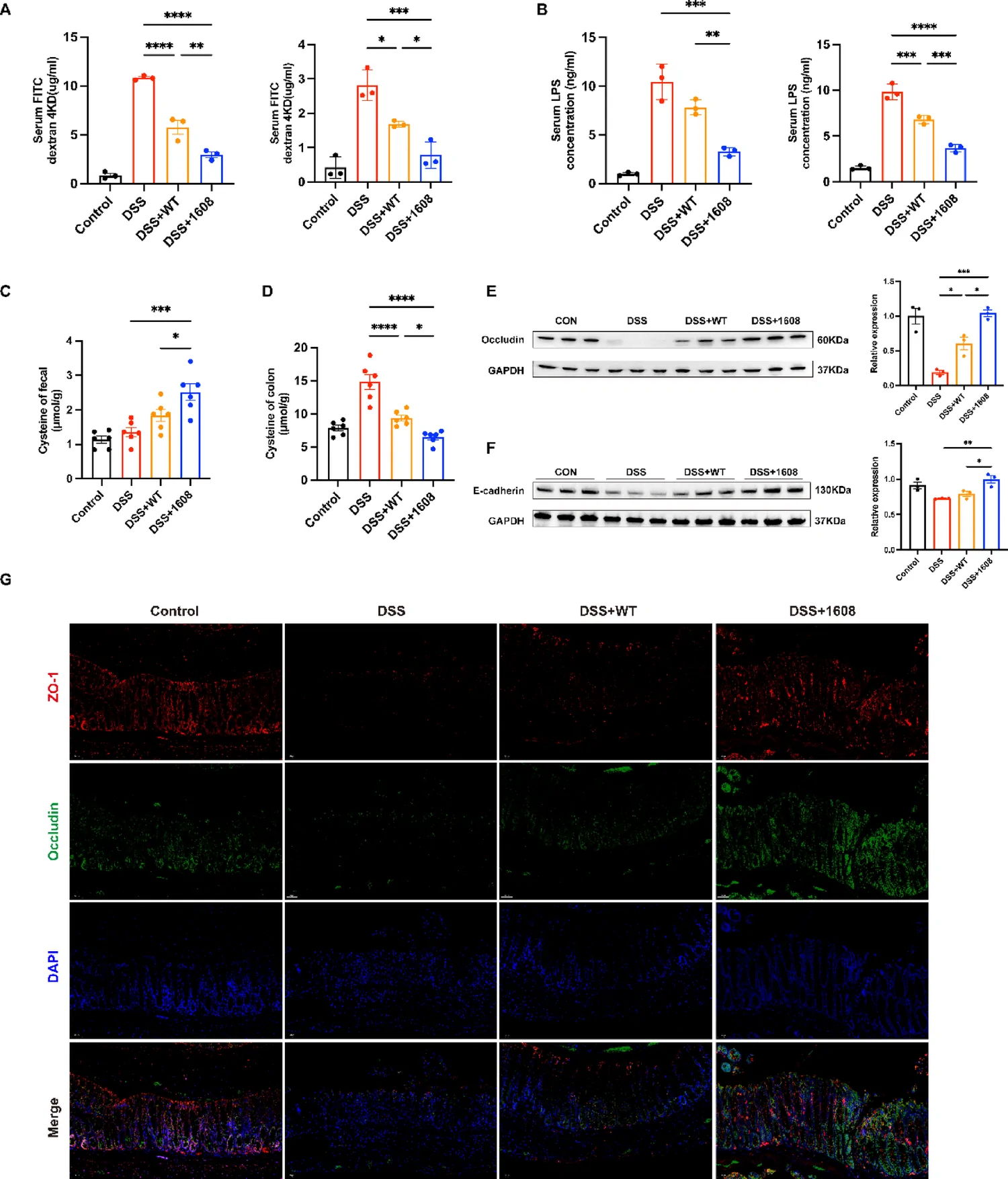
BV1608 reduces intestinal cysteine availability and restores gut barrier integrity. **A** Serum levels of FITC-dextran 4KD (FD4) in mice from different treatment groups. (Left- acute colitis model; Right-chronic colitis model). **B** Serum levels of lipopolysaccharide (LPS) in mice from different treatment groups. (Left- acute colitis model; Right-chronic colitis model). **C** Fecal cysteine levels in mice among different treatment groups. **D** Cysteine levels in the intestinal epithelium of the different treatment groups. **E–F** Group comparison of western blot analysis results for gut barrier–associated proteins Occludin and E-cadherin with GAPDH serving as internal control. **G** Representative immunofluorescence staining images of colon sections to indicate the expression of tight junction proteins, including ZO-1 and Occludin after different treatments. (Scale bar:50 μm). Data are presented as mean values ± SEM. Statistical analysis was performed using one-way ANOVA followed by Tukey’s multiple comparison post hoc tests. *P < 0.05, **P < 0.01, ***P < 0.001 and ****P < 0.0001 (*n* = 3 biologically independent samples for A, B, E and F, *n* = 6 biologically independent samples for C and D)

The impairment of the intestinal barrier represents a key pathological event in the initiation and progression of intestinal inflammation [21]. The integrity of the gut barrier was evaluated by measuring permeability markers. Serum FITC-dextran 4KD (FD4) levels, elevated in DSS models, were decreased by both bacterial treatments, with the effect being more pronounced in the BV1608 group (Fig. 4A). This enhanced restoration of barrier function was also corroborated by a significant decrease in serum lipopolysaccharide (LPS) levels specifically in the BV1608-treated mice (Fig. 4B). We subsequently assessed the cysteine-metabolizing capacity of BV1608 in the gut. Quantification of cysteine levels in intestinal epithelial cells and fecal samples revealed a significant decrease in intracellular cysteine and a concurrent increase in fecal cysteine in the BV1608-treated group, respectively (Fig. 4C–D). We investigated the expression of key tight junction proteins, Occludin and E-cadherin, as indicators of gut barrier integrity. Our results showed that DSS-induced colitis significantly impaired their expression, and this impairment was reversed by treatment with BV1608 (Fig. 4E–F). Consistent with our previous findings, immunofluorescence analysis of these tight junction proteins further confirmed the protective effects of both BV-WT and BV1608 on the intestinal barrier, with BV1608 exhibiting a markedly superior efficacy (Fig. 4G).

### Administration of engineered Bacteroides reshaped the CD4^+^ T cell-associated immune microenvironment in the murine colon

**Fig. 5:**
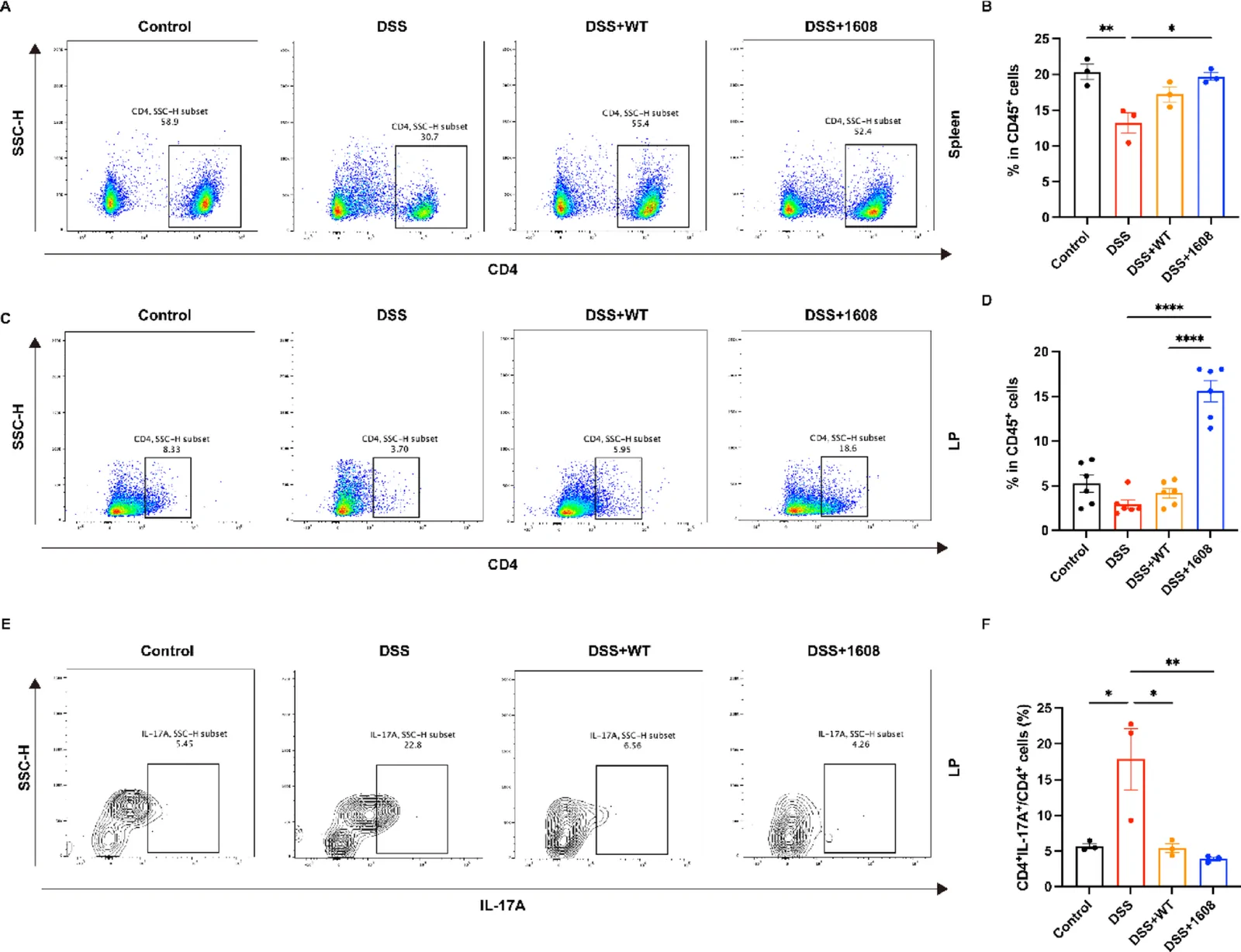
BV1608 reshapes the colonic immune microenvironment by suppressing Th17 cell differentiation. **A–B** The percentage of CD4^+^ T cells among total immune cells (CD45^+^ cells) within the spleen was detected by flow cytometry and statistically analysed. **C–D** The percentage of CD4^+^ T cells among total immune cells (CD45^+^ cells) within the LP immune cells was detected by flow cytometry and statistically analysed. **E–F** The percentage of IL-17 A Th17 cells among total CD4^+^ T cells within the LP immune cells was detected by flow cytometry and statistically analysed. Data are presented as mean values ± SEM. Statistical analysis was performed using one-way ANOVA followed by Tukey’s multiple comparison post hoc tests. *P < 0.05, **P < 0.01 and ****P < 0.0001 (*n* = 3 biologically independent samples for Band F, n=6 biologically independent samples for D)

Before applying BV1608 intervention in colitis, we administered the engineered bacteria via continuous oral gavage to mice under non-inflammatory conditions to observe whether it would affect the intestinal immune microenvironment. After one week of continuous gavage, LPMCs were isolated, and flow cytometry was used to assess changes in the intestinal immune cell landscape induced by BV1608 (Supplementary Fig. 1). We found that BV1608 did not significantly perturb the intestinal immune microenvironment under homeostatic conditions(Control and BV1608). Given the pivotal role of the CD4^+^ T cell-mediated immune microenvironment in the pathogenesis of ulcerative colitis, we therefore conducted a flow cytometric analysis of CD4^+^ T cells from both spleen (Fig. 5A–B) and gut-localized (colon lamina propria) compartments (Fig. 5C–D). In mice treated with DSS alone, the percentage of CD4^+^ T cell decreased in spleen relative to that in control mice. Conversely, BV1608 treatment led to a marked increase in CD4⁺ T cell proportion in both the spleen and the lamina propria, particularly in the latter—a phenomenon likely attributable to the oral gavage model that facilitates localized treatment of the intestine with the engineered bacteria. Notably, among the expanded CD4⁺ T cell population, the pro-inflammatory Th17 subset is of particular pathophysiological relevance. Th17 cells, as a key pro-inflammatory T helper subset, are centrally implicated in the pathogenesis of colitis by driving intestinal inflammation through the production of cytokines such as IL-17 and IL-22 [22]. The infiltration of Th17 cells into the lamina propria induced by DSS was significantly reduced by BV1608 treatment (Fig. 5E–F). To determine whether this reduction in Th17 cell proportions translates into functional immunological differences, we measured the levels of Th17-associated cytokines. Our results demonstrated that BV1608 treatment significantly reduced both IL-17 A and IL-22 levels, indicating a functional suppression of Th17-related inflammatory responses (Supplementary Fig. 2). Collectively, these data demonstrated that treatment with the engineered bacteria fundamentally reshaped the gut immune microenvironment by attenuating the differentiation of CD4⁺ T cells towards Th17 cells.

### Cystine/Cysteine restriction suppresses Th17 differentiation in an ATF6-dependent manner and is associated with BATF2 upregulation

**Fig. 6:**
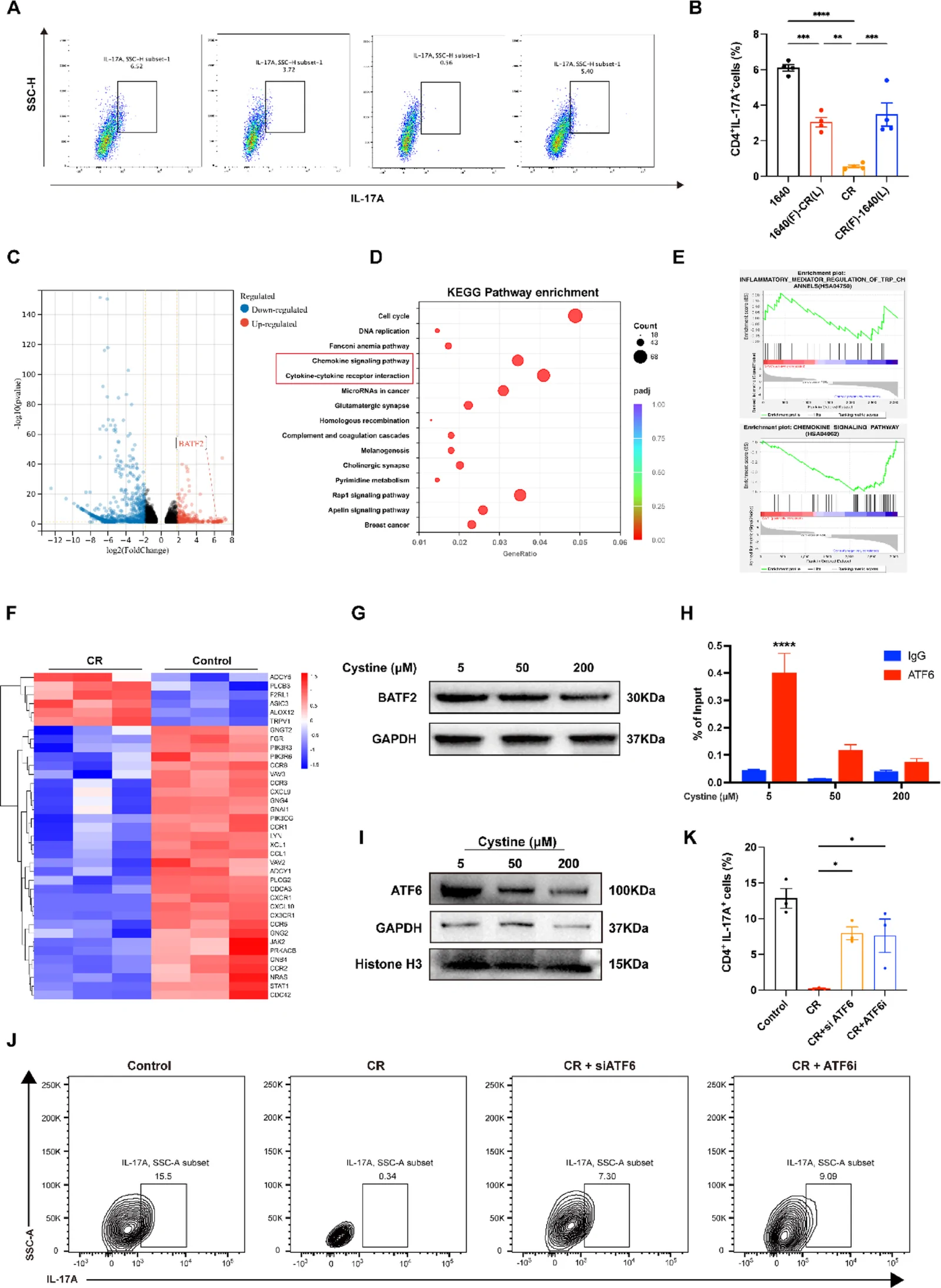
Cystine limitation inhibits Th17 cell differentiation through an ATF6-dependent mechanism and is accompanied by increased BATF2 expression. Naïve CD4⁺ T cells from healthy donors were polarized under Th17 conditions for 5 days. **A–B** The percentage of IL-17 A Th17 cells among total CD4^+^ T cells cultured in different medium was detected by flow cytometry and statistically analysed. **C** The volcano plot depicted the top 50 differentially expressed genes, selected based on a |log2 fold change| > 0.585 and a P value < 0.05 (*n* = 3 per group). **D** KEGG pathway enrichment analysis of differentially expressed genes. **E** GSEA revealed two pathways associated with inflammatory in the cystine-restricted CD4^+^ T cells. **F** Heatmap of differentially expressed genes associated with the inflammatory mediator regulation of TRP channels and the chemokine signaling pathway. **G** The expression of BATF2 protein in Naïve CD4⁺ T cells was analyzed by Western blotting, using GAPDH as control, after the cells were cultured in a gradient of cysteine-restricted medium for 2 days. **H** ChIP-qPCR assays were performed to assess ATF6 binding at candidate sites within the BATF2 promoter under varying cystine concentrations. **I** Western blot analysis of ATF6 nuclear translocation induced by cystine restriction. **J–K** Representative flow cytometry plots and quantification of CD4⁺ IL-17 A⁺ T cells in Control, CR (cystine restriction), CR + siATF6 (CR combined with ATF6 siRNA transfection), and CR + ATF6i (CR combined with ATF6 inhibitor treatment) groups. Group 1640: Cultured in complete RPMI 1640 medium throughout; Group 1640(F)-CR(L): Cultured in complete medium for the first half, then switched to cystine-restricted (CR) medium; Group CR: Cultured in cystine-restricted medium throughout; Group CR(F)-1640(L): Cultured in cystine-restricted medium for the first half, then switched to complete medium. (F): First half; (L): Second half. Data are presented as mean values ± SEM. Statistical analysis was performed using one-way or two-way ANOVA. *P < 0.05, **P < 0.01, ***P < 0.001, ****P < 0.0001 (*n* = 3 biologically independent samples for H and K, n=4 biologically independent samples for B)

Based on these in vivo findings, we aimed to directly investigate the role of cystine/cysteine in restricting the differentiation of CD4⁺ T cells into Th17 cells using an in vitro approach. We assessed the differentiation of CD4^+^ T cells into Th17 cells by flow cytometry, using PBMCs cultured under Th17-polarizing conditions in a cystine-restricted medium (CR). We found that Th17 cell differentiation from CD4^+^ T cells was suppressed regardless of the initial order of media exposure, as long as the culture included a cystine-restriction phase. Furthermore, the suppression of CD4^+^ T cell differentiation was most pronounced when cells were maintained under continuous cystine restriction throughout the entire culture period (Fig. 6A–B). To elucidate the mechanistic basis of CD4^+^ T cell differentiation modulation by cystine availability, we conducted transcriptomic analysis via RNA sequencing on cells from both the control (1640 medium) and CR (cystine-restricted medium) groups. Our analysis identified 3074 differentially expressed genes (DEGs; 1246 up-regulated and 1828 down-regulated). BATF2 was among the upregulated genes identified from the transcriptomic screen and has previously been reported to suppress Th17-associated inflammatory responses [23]. (Fig. 6C). The Kyoto Encyclopedia of Genes and Genomes (KEGG) enrichment analysis revealed significant enrichment in multiple pathways, including the chemokine signaling pathway and cytokine-cytokine receptor interaction pathway, which are closely associated with inflammation (Fig. 6D). Gene set enrichment analysis (GSEA) further confirmed the downregulated chemokine signaling pathway, along with the upregulated inflammatory mediator regulation of TRP channels (Fig. 6E). Additionally, the expression profiles of the DEGs associated with these pathways are displayed in a heatmap (Fig. 6F). Furthermore, we validated the upregulation of BATF2 expression in CD4⁺ T cells under specific cystine-restricted culture conditions (Fig. 6G). Bioinformatic analysis using the JASPAR database revealed that the BATF2 upstream promoter region contains multiple binding sites for the transcription factor ATF6 (Supplementary Fig. 3A). Subsequent ChIP-qPCR analysis revealed that the binding of ATF6 to Site 1 was significantly enriched under cystine-restricted conditions (Fig. 6H). We hypothesized that cystine restriction promotes the nuclear translocation of ATF6, thereby enhancing its binding to the BATF2 promoter. Indeed, nuclear/cytoplasmic fractionation assays confirmed a significant accumulation of ATF6 in the nucleus under cystine-restricted conditions (Fig. 6I). To further determine whether ATF6 is functionally involved in cystine restriction-mediated suppression of Th17 differentiation, we performed ATF6 loss-of-function experiments under CR conditions. ATF6 was suppressed either by siRNA-mediated knockdown (Supplementary Fig. 3B) or by pharmacological inhibition. Flow cytometry analysis showed that CR markedly reduced the percentage of CD4⁺IL-17 A⁺ T cells compared with the control group. Notably, both CR + siATF6 (CR combined with ATF6 siRNA transfection) and CR + ATF6i (CR combined with ATF6 inhibitor treatment) partially restored the proportion of CD4⁺IL-17 A⁺ T cells under cystine-restricted conditions (Fig. 6J–K).

### *B. vulgatus* BV1608 modulate gut microbiota composition in DSS-induced colitis mice

**Fig. 7:**
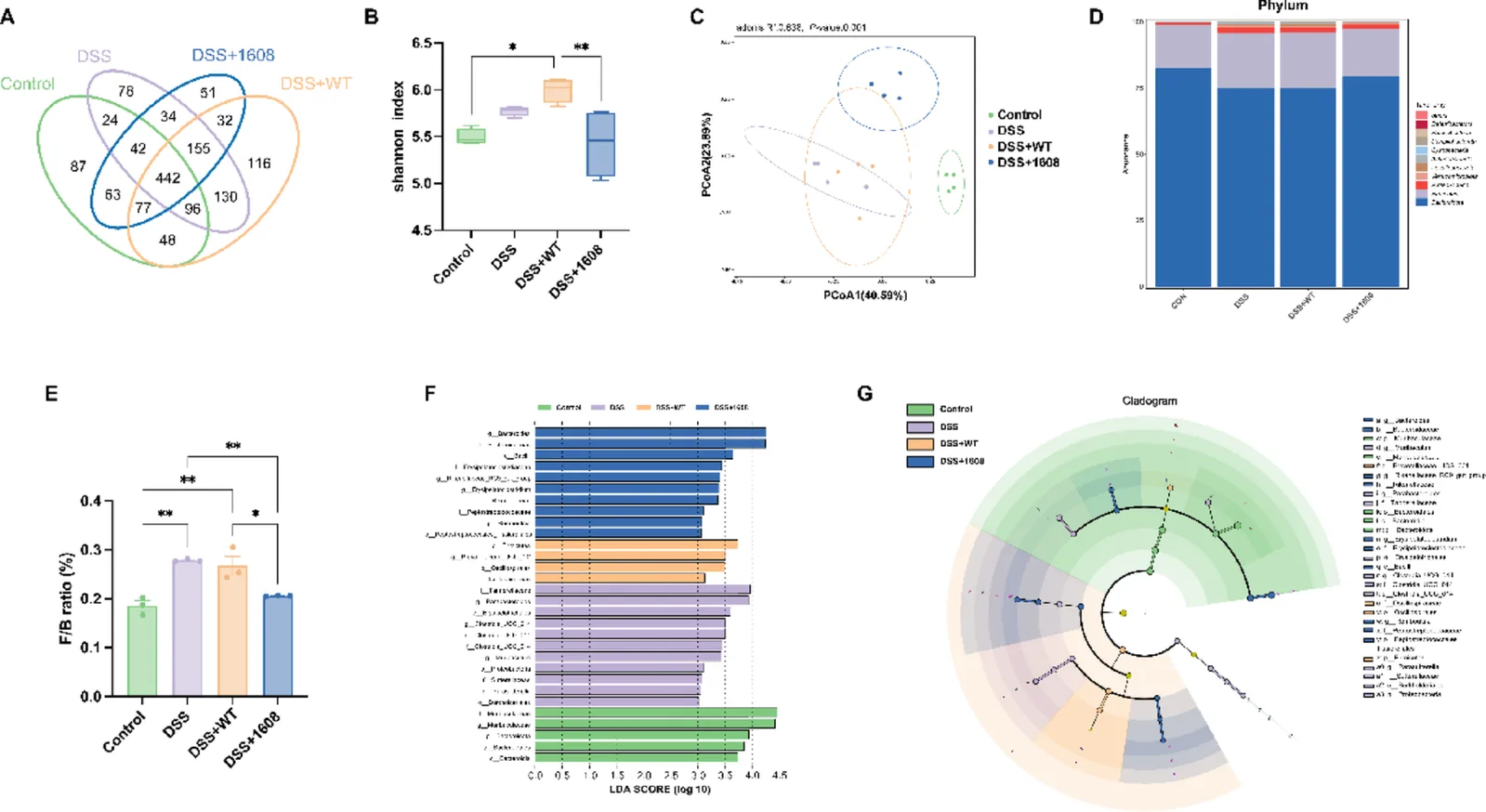
BV1608 treatment modulates gut microbiota composition and reverses dysbiosis in colitis mice. **A** Venn diagram showing the overlap of the OTUs identified in the gut microbiota among the four groups. **B** The Shannon index of the fecal microbiota among the four groups. **C** The principal coordinates analysis (PCoA) plot of the gut microbiota based on the Bray–Curtis metric (R^2^:0.638;* P*:0.001). **D** The top 10 microbiota taxa found at the phylum level. **E** The ratio of Firmicutes / Bacteroidetes (F/B) among the four groups. **F–G** The distribution histogram based on LDA (LDA > 3). Data are presented as mean values ± SEM. Statistical analysis was performed using one-way ANOVA followed by Tukey’s multiple comparison post hoc tests. *P < 0.05 and **P < 0.01 (*n* = 4 biologically independent samples for B, n=3 biologically independent samples for E)

Given the pivotal role of gut microbiota in maintaining intestinal homeostasis [24], analyzing whether the administration of *B. vulgatus* BV1608 could reverse colitis-induced dysbiosis was crucial for a comprehensive and systematic understanding of this engineered strain’s therapeutic strategy. The 16 S rRNA sequencing was used to test the changes of gut microbiota. The average numbers of operational taxonomic units (OTUs) and OTUs shared by the different groups were visualized using a Venn diagram (Fig. 7A). Compared to the control, DSS induced a slight but non-significant increase in the Shannon index, indicating a trend toward higher microbial richness and diversity. This increase was significantly amplified by the WT strain but was abolished in the DSS + 1608 group. (Fig. 7B). Then, the β-diversity analysis was carried out to generate a PCoA plot to evaluate the extent of the similarity between the microbial communities. DSS intervention greatly affected the overall microbial composition, compared with that in the Control group, and the oral BV1608 administration led to a significant shift in the gut microbial profile (PERMANOVA, R² = 0.638, *P* = 0.001) (Fig. 7C). BV1608 administration significantly reversed the DSS-induced compositional shifts in Firmicutes and Bacteroidetes, ultimately restoring the diminished F/B ratio to near-normal levels (Fig. 7D–E). Microbial taxa with LDA scores > 3 was visualized in Fig. 7F–G. The DSS + 1608 group showed enrichment of 10 discriminative taxa, while 5, 11 and 4 taxa were predominant in the Control, DSS and DSS + WT groups, respectively.

## Discussion

Ulcerative colitis is characterized by persistent inflammation and epithelial damage, driven by a complex interplay between the gut microbiome and immune dysregulation [25]. In this study, we developed a cysteine-auxotrophic and metabolically enhanced strain of *Bacteroides vulgatus*, BV1608, and demonstrated its therapeutic efficacy in both acute and chronic DSS-induced colitis models. Our findings suggest that targeted microbial cysteine depletion alleviates intestinal inflammation, at least in part, by limiting pathogenic Th17 differentiation through an ATF6-dependent mechanism. In parallel, cystine restriction was associated with BATF2 upregulation and increased ATF6 binding at the BATF2 promoter, implicating BATF2-associated transcriptional regulation as a potential downstream mechanism. These results highlight the therapeutic potential of rationally engineered probiotics and provide evidence for a metabolite–immune regulatory model relevant to inflammatory bowel disease.

Cysteine plays a vital role in the biosynthesis of glutathione (GSH), which functions as the most abundant and crucial antioxidant in cells [26]. It is widely observed that the colonic mucosa in active ulcerative colitis exhibits a marked reduction in glutathione levels, accompanied by a concomitant increase in oxidative stress [27–29]. Cysteine depletion impairs GSH biosynthesis, thereby promoting ferroptosis and exacerbating ulcerative colitis [30]. While this may initially appear counterintuitive to our original design rationale for the engineered strain, from an immunological perspective, cysteine is a critical prerequisite for T cell activation and differentiation [31]. Consequently, the modulation of cysteine availability serves as a potent metabolic checkpoint. Our data demonstrated that BV1608-mediated cysteine restriction significantly suppressed Th17 differentiation in the lamina propria. This suggests that by limiting the local cysteine pool, BV1608 effectively raises the metabolic threshold for T cell activation, thereby dampening the aberrant Th17 response characteristic of colitis. Importantly, our additional safety assessment showed that BV1608 administration did not significantly alter colonic GSH or MDA levels under the tested conditions, suggesting that the engineered strain did not impair intestinal glutathione homeostasis or exacerbate oxidative stress. This is likely because the colonic epithelium and mucosal immune cells can maintain intracellular GSH pools by utilizing basolateral cystine uptake from the systemic circulation, which may partially compensate for the restriction of luminal cysteine availability.

Our study demonstrated the therapeutic potential of an engineered, cysteine-auxotrophic strain of *Bacteroides vulgatus*, BV1608, in murine models of colitis and delineates its underlying immune-metabolic mechanism. First, we enhanced the cysteine-scavenging capacity of BV1608 by chromosomally integrating the *E. coli cyuP* gene, which encodes a high-affinity cysteine transporter. In a cysteine-supplemented specific medium, the engineered strain demonstrated enhanced proliferative capacity and improved cysteine uptake efficiency without systemic toxicity. Furthermore, the safety profile of utilizing metabolic competition as a therapeutic strategy warrants critical examination. It is well-established that severe cysteine deprivation can precipitate glutathione exhaustion, thereby inducing ferroptosis and potentially exacerbating intestinal inflammation [32].

In both acute and chronic DSS-induced colitis, oral administration of BV1608 significantly alleviated disease severity, restored intestinal barrier integrity, and reduced systemic inflammation. In this process, the wild-type *B. vulgatus* also exerted a protective effect on intestinal barrier function, which is consistent with previous reports [33]; however, this effect was markedly less pronounced than that of the engineered strain. In addition, under non-colitic conditions, BV1608 administration did not induce significant changes in the examined intestinal immune cell profiles, suggesting that BV1608 does not overtly disturb immune homeostasis in the absence of inflammatory stimulation. Mechanistically, BV1608-mediated restriction of local cysteine availability reshaped the intestinal immune microenvironment, notably suppressing the differentiation of pathogenic Th17 cells in the lamina propria. Through in vitro cystine-restriction assays coupled with transcriptomic profiling, we found that cystine availability influences CD4⁺ T cell differentiation and is associated with activation of ATF6 signaling and increased BATF2 expression. Together with the ATF6 loss-of-function data, these findings support a functional role for ATF6 in cystine restriction-mediated suppression of Th17 differentiation, while implicating BATF2 as a potential downstream transcriptional node. Rather than establishing a fully defined linear pathway, our data provide evidence for a metabolite–immune regulatory mechanism through which engineered microbial cysteine sequestration may modulate pathogenic T cell responses in intestinal inflammation.

ATF6 (Activating Transcription Factor 6), a key sensor of the unfolded protein response (UPR) that is activated upon endoplasmic reticulum (ER) stress and translocates to the nucleus to regulate genes related to protein homeostasis, has also been implicated in immune modulation beyond its canonical role [34–35]. In our study, transcriptomic and bioinformatic analyses revealed that cystine restriction upregulates BATF2 expression, and the BATF2 promoter contains multiple predicted ATF6-binding sites, suggesting a potential ATF6-associated regulatory relationship with BATF2 expression. Notably, BATF2 (Basic Leucine Zipper ATF-Like Transcription Factor 2), a member of the AP-1/ATF superfamily, has recently been identified as a negative regulator of pro-inflammatory T cell responses, particularly through inhibiting Th17 cell differentiation and attenuating intestinal inflammation by interfering with the IL-23/IL-17 axis [36].

Based on these findings, We propose that BV1608, by sequestering extracellular cysteine in the inflamed gut, creates a localized cystine-restricted microenvironment that suppresses pathogenic Th17 differentiation at least partly through ATF6-dependent signaling. In parallel, cystine restriction was associated with BATF2 upregulation, increased ATF6 nuclear accumulation, and enriched ATF6 binding at the BATF2 promoter, suggesting BATF2 as a candidate downstream transcriptional node. Nevertheless, BATF2-specific loss-of-function or rescue experiments were not included in the present study because definitive BATF2 perturbation and rescue experiments would require additional optimization of BATF2 knockdown or overexpression in primary CD4⁺ T cells under Th17-polarizing and cystine-restricted conditions, as well as careful control of transfection efficiency, cell viability, and differentiation bias. Therefore, future work will be required to further define the linearity and indispensability of the proposed ATF6–BATF2–Th17 regulatory axis.

In conclusion, we demonstrated that engineered *B. vulgatus* ameliorated experimental colitis by creating a local cystine-restricted microenvironment. This metabolic alteration activates ATF6 and is associated with BATF2 upregulation in CD4⁺ T cells, thereby suppressing pathogenic Th17 differentiation. Our findings provide a mechanistically informed basis for metabolism-targeted probiotics and suggest that microbial cysteine sequestration may represent a promising strategy for modulating pathogenic Th17 responses in ulcerative colitis.

## Supplementary Information

Below is the link to the electronic supplementary material.

Supplementary Material 1

## Data Availability

16 S rRNA gene sequencing data have been deposited into the NCBI Sequence Read Archive (SRA) database with the accession number PRJNA1399114. RNA-sequencing data have been deposited in NCBI SRA database with the accession number PRJNA1399064.
