# Supplementary material for: Depleting luminal cysteine with engineered bacteroides vulgatus alleviates experimental colitis by suppressing Th17 differentiation through an ATF6-dependent mechanism
Source: Inflamm Res. 2026 Sep 5;75(1):200. doi: 10.1007/s00011-026-02341-3 (PMC13546354; doi:10.1007/s00011-026-02341-3)
Supplement: Supplementary file 1 [file 11_2026_2341_MOESM1_ESM.docx]

**Supplementary Figure1.** Effects of oral administration of BV1608 on intestinal immune cells under non-inflammatory conditions. Representative flow cytometry plots (A) and statistical results (B) of CD4^+^ T cells, CD8^+^ T cells, B cells, macrophages, and DCs in the intestinal lamina propria.

**Supplementary Figure2.** Serum levels of Th17-associated cytokines in DSS-induced colitis. Concentrations of (A) IL-17A and (B) IL-22 in the serum of mice across different experimental groups. Data are presented as mean ± SEM. Statistical significance was determined by one-way ANOVA followed by Tukey's post hoc test. *p < 0.05, **p < 0.01 and ****P < 0.0001.

**Supplementary Figure3.** (A) Schematic representation of putative ATF6 binding site in promoter region of BATF2. (B) qPCR was used to detect the expression level of ATF6. Data are presented as mean ± SEM. Statistical significance was determined by one-way ANOVA followed by Tukey's post hoc test. ***p < 0.001.
